# Successful Long-Term Management of Canine Superficial Necrolytic Dermatitis With Amino Acid Infusions and Nutritionally Balanced Home-Made Diet Modification

**DOI:** 10.3389/fvets.2020.00028

**Published:** 2020-01-31

**Authors:** Jared A. Jaffey, Robert C. Backus, Megan Sprinkle, Catherine Ruggiero, Sylvia H. Ferguson, Kate Shumway

**Affiliations:** ^1^Department of Small Animal Medicine and Surgery, College of Veterinary Medicine, Midwestern University, Glendale, AZ, United States; ^2^Department of Veterinary Medicine and Surgery, Veterinary Health Center, University of Missouri, Columbia, MO, United States; ^3^Department of Pathology and Population Medicine, College of Veterinary Medicine, Midwestern University, Glendale, AZ, United States

**Keywords:** hepatocutaneous syndrome, hypoaminoacidemia, nutrition, vitamin D, vitamin A, lysine

## Abstract

A 10-year old, castrated male, Bichon Frise with a history of hyperadrenocorticism and intrahepatic portal vein hypoplasia was diagnosed with superficial necrolytic dermatitis (SND). The dog exhibited thick crusts on the chin, muzzle, prepuce, and paws. In addition, the dorsal surfaces of all paws were erythematous while the palmar/plantar surfaces were hyperkeratotic, hardened, and painful. The dog was treated with intravenous amino acid infusions (AAI), raw egg yolks, as well as zinc and omega-3 fatty acid oral supplements. The dog required AAI once every 2–3 weeks because this coincided with recrudescence of painful skin lesions. The dog was subsequently diagnosed with diabetes mellitus. A consult with the Nutrition Service was pursued 220 days after the original SND diagnosis because of concern for feeding raw eggs and for malnutrition since appetite was variable, muscle condition was reduced, and greater than 50% of ingested calories were from foods that were not nutritionally complete. There was also concern regarding the variability of the diet and the impact it would have on the management of diabetes mellitus. The diet was prepared by the dog owner according to a provided recipe and presented twice daily. The diet was rich in high quality protein and fat. All other treatments including medications, supplements, and bathing schedule remained unchanged at the time of diet modification. The dog was subclinical for SND associated clinical signs approximately 3 weeks after the diet modification, which also coincided with the last AAI. The AAI was postponed and was next administered 7 weeks later (i.e., 10 weeks from the previous infusion). The dog remained subclinical for SND related clinical signs and continued to receive AAI once every 10–12 weeks until he was euthanized 718 days later for complications related to severe multi-drug resistant, skin infections. In conclusion, this report highlights a novel role for nutritionally balanced home-made diets designed by a board-certified veterinary nutritionist could substantially increase time interval between AAI and outcome in dogs with SND.

## Background

Superficial necrolytic dermatitis (SND) also known as hepatocutaneous syndrome, is an uncommon skin disorder in dogs. The most common clinical manifestation of SND in dogs is the development of painful and variable pruritic skin lesions involving the footpads, peri-ocular/oral/anal or genital regions, and pressure points on the trunk and limbs (1).

Long-term prognosis for dogs with SND is generally poor with reported survival times of 6–12 months (2). Currently, administration of intravenous amino acids is suggested as the most effective palliative form of therapy (1, 2). Repeated amino acid infusions (AAI) are costly, however, and contribute to increased morbidity with repeated central-line catheters or indwelling venous access ports. These treatments are often cost-prohibitive for dog owners and can result in the decision of euthanasia or death as result of procedure complications including phlebitis, fibrosis of jugular vessels, and catheter associated thromboembolism.

In this article we report, for the first time, the adjunctive role of a prescription home-cooked diet containing quality protein for the long-term management of a dog with SND that yielded an improvement in clinical signs and a reduction in the frequency of AAI.

## Case Presentation

A 10-year old male castrated Bichon Frise was presented to the University of Missouri Veterinary Health Center for evaluation of hyporexia for 2 weeks. Past pertinent history included histopathologic confirmation of intrahepatic portal vein hypoplasia at a young age and hyperadrenocorticism diagnosed at 9 years old. Physical examination abnormalities included a “pot belly” appearance, hepatomegaly, and bilaterally symmetrical, noninflammatory truncal hypotrichosis. There were no other dermatologic abnormalities.

**Figure 1:**
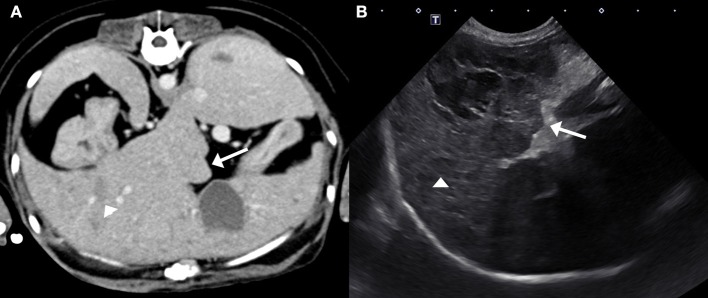
Post-contrast computed tomography image **(A)** and ultrasound image **(B)** of the liver. The hepatic margins are rounded (arrows) and the liver is mottled with ill- defined hypoattenuating/hypoechoic nodules (arrow heads). These nodules are better defined on the ultrasound image creating the classical “Swiss-cheese” or “honeycomb” appearance of the liver associated with SND.

The following day a fasted ammonia was performed and was within the reference interval (18.7 μmol/L; reference range 0–45 μmol/L). Next, a computed tomography (CT) of the head and the abdomen were performed to investigate the cause for hyporexia, which was a concern because the dog had untreated hyperadrenocorticism and had historically been polyphagic. Differential diagnoses considered included pituitary macroadenoma or intra-abdominal disease. The CT did not reveal any abnormalities in the brain but did show the liver was diffusely mottled and hyperattenuating on pre-contrast images, with rounded, nodular margins. Following administration of intravenous contrast, the mottled appearance of the liver was exaggerated (Figure 1A). On ultrasonographic imaging, the liver had rounded margins and contained numerous hypoechoic nodules (Figure 1B). Ultrasound-guided needle core biopsies of the liver were subsequently performed and submitted for histopathology, aerobic/anaerobic bacterial culture, and copper quantification. The dog was treated with oral amoxicillin/clavulanic acid (Clavamox, Zoetis Inc., Kalamazoo, MI; 15.6 mg/kg twice daily) until results from the liver biopsy returned. These results showed mild lymphoplasmacytic and suppurative hepatitis with severe edema or glycogen associated cell swelling. The diagnostic level of copper was within the normal reference interval and the bacterial culture was negative.

The dog was presented 3 weeks later for evaluation of persistent hyporexia and an acute onset of lethargy. Physical examination remained unchanged. In order to investigate the unexplained hyporexia and lethargy, a magnetic resonance imaging of the brain was performed in order to definitively rule out intra-cranial disease; the study was unremarkable. Next, a gastroduodenoscopy was performed with biopsies of the stomach and duodenum, which were all unremarkable. Medical management at the time of discharge included oral ursodiol (URSO 250, Aptalis Pharma Inc., Bridgewater, NJ; 12.5 mg/kg once daily) and trilostane (Vetoryl, Dechra Veterinary Products, Overland Park, KS; 1.6 mg/kg twice daily).

**Figure 2:**
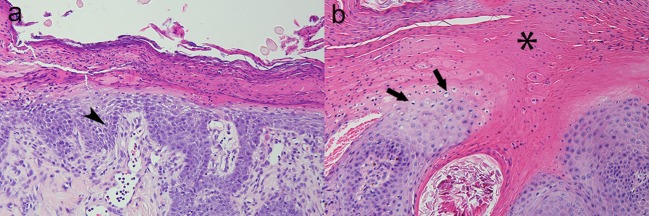
Canine; paw pad. Photomicrographs illustrate a “red, white, and blue” pattern to the epidermis typical of superficial necrolytic dermatitis (also known as hepatocutaneous syndrome). The characteristic lesions are parakeratosis (“red,” asterisk), superficial keratinocyte vacuolar degeneration due to accumulation of intracellular and intercellular edema (“white,” arrows), and basilar epidermal hyperplasia (“blue,” arrow head). Hematoxylin and eosin stain. Magnification for **(a)** =200x, **(b)** =400x.

The dog was evaluated 14 days later to assess the medical management of hyperadrenocorticism. The clinical history (e.g., resolution of polyuria/polydipsia) and ACTH-stimulation diagnostic results (pre-ACTH stimulated serum cortisol: 2.5 μg/dL; 1 h post-ACTH stimulated serum cortisol: 3.6 μg/dL) indicated adequate management of hyperadrenocorticism. Interestingly, the dog's appetite improved but severe pruritus, especially of the paws, was noted. Dermatologic changes consisted of thick crusts on the chin, muzzle, prepuce, and paws. In addition, the dorsal surfaces of all paws were erythematous while the pawpads were hyperkeratotic, hardened, and painful. Four, 4 mm punch skin biopsies were obtained from left hind hock and metacarpal pad, right hind metacarpal pad, and left front metacarpal pad and revealed marked epidermal hyperplasia that measured 2–3 microns thick and had a laminated, “red, white, and blue” appearance that is classically observed in cases of SND. More specifically, orthokeratotic and parakeratotic hyperkeratosis (“red”) with extensive serocellular crusting, swollen and pale superficial keratinocytes due to intracellular and intercellular edema (“white”) and mild basal cell hyperplasia (“blue”) were observed. The superficial dermis was expanded by increased clear space and featured ectatic lymphatics (edema), ectatic, hyperemic vessels which contained increased numbers of marginating neutrophils, and was infiltrated by low numbers of lymphocytes and plasma cells (Figure 2). The nature of the disease underlying SND in this dog was unknown, but suspected to be due to an underlying severe hepatopathy of unknown etiology in combination with metabolic disturbances associated with hyperadrenocorticism. The pancreas appeared normal on contrast enhanced CT of the abdomen.

Following confirmation of SND, the dog was presented for initiation of therapy (day 1). An amino acid solution (TrophAmine®, Braun Medical Inc., trophamine 10%) was administered intravenously via a central catheter at a rate of 14 ml/h (2.4 mL/kg/h) over 10 h (dosage 25 mL/kg). Additional therapies initiated at that time included oral zinc supplement (NutriVed ZinPro; 2.6 mg/kg once daily), omega-3-fatty acid (Nutramax Welactin 3; omega-3 fatty acids 52.6 mg/kg, eicosapentaenoic acid 27.2 mg/kg, docosahexaenoic acid 17.5 mg/kg once daily), and two raw egg yolks daily. The dog was also bathed with Douxo Chlorohexidine PS shampoo (Douxo, Sogeval Laboratories Inc., Coppell, TX) once per week. Treatment with trilostane and ursodiol remained unchanged. At that time the dog was fed a combination of human food and Hill's® Science Diet® Adult Sensitive Stomach & Skin dog food. AAI (25 mL/kg over 10–12 h) were administered once per week for 4 weeks. The dog was presented on day 31 and the owner reported there had been a gradual but substantial clinical improvement. Dermatologic examination revealed minimal crusting of the skin around the chin, paws, and prepuce.

Attempts were made over the next 2 months to decrease the frequency of AAI but recrudescence of clinical signs (e.g., hyporexia, lethargy, and erythema/crusts of the skin) necessitated treatments every 2–3 weeks. On day 91 the dog was diagnosed with diabetes mellitus and medical management (Vetsulin; 0.32 units/kg administered subcutaneously twice daily) was initiated. Subsequent blood glucose curve assessments performed in-hospital on two occasions over the next 6 weeks combined with weight gain, lack of polyuria, polydipsia, and polyphagia indicated the dog's diabetes mellitus had been adequately managed.

A consult with the Nutrition Service at the University of Missouri Veterinary Health Center was pursued on day 220 because there was concern for risks involved with feeding raw eggs and for malnutrition since appetite was variable. Muscle condition was reduced and greater than 50% of ingested calories were from foods that were not nutritionally complete. There was also concern regarding the variability of the diet and the impact it would have on the management of diabetes mellitus. All other forms of medical management including trilostane, insulin, ursodiol, zinc/omega-3 fatty acid supplementation, and bathing schedule remained unchanged. The only change to medical management was introduction of a nutritionally complete and balanced diet (Table 1). At the time of the consultation, body weight, body condition and muscle condition scores were 6.2 kg, 5–6/9 (4–5/9 ideal), and 2/3 (3/3 normal), respectively. The diet was prepared by the owner according to a provided recipe and presented twice daily to coincide with insulin injections. The diet was rich in amino acids and polyunsaturated fatty acids. The protein sources are regarded as highly digestible for dogs after cooking (Table 1).

**Table 1:** Ingredients and nutrient contents of home-prepared diet prescribed for adjunctive management of necrolytic migratory erythema in a dog with hyperadrenocorticism, intrahepatic portal vein hypoplasia, and diabetes mellitus.

	**Homemade diet**		**NRC RA^a^**
**Food/Nutrients**	**Per day**	**Per 1,000 kcal**	**Per 1,000 kcal**
Chicken breast (skinless, boneless, stewed)^b^ (g)	85	207	–
Large whole egg (cooked)^b^ (g)	61	148	–
Large egg yolk (cooked)^b^ (g)	17	41	–
Sweet potato (baked in skin, without salt)^b^ (g)	134	326	–
Cold-water, marine, fish oil^c^ (g)	1.8	4.4	–
Vitamin-mineral supplement^d^ (g)	6.1	15	–
**Proximates**			
Protein (g)	38	91	25
Protein (% metabolizable energy)	37	–	–
Fat (g)	16	38	14
Fat (%metabolizable energy)	35	–	–
Carbohydrate (g)	29	70	–
Carbohydrate (%metabolizable energy)	28	–	–
Fiber (g)	4.4	11	–
Ash (g)	3.5	–	–
Moisture (g)	214	518	–
**Essential amino acids**			
Tryptophan (g)	0.6	1.3	0.4
Threonine (g)	1.7	4.0	1.0
Isoleucine (g)	2.0	4.7	0.9
Leucine (g)	2.9	7.0	1.7
Methionine (g)	1.0	2.5	0.8
Cystine (g)	0.6	1.4	0.8
Phenylalanine (g)	1.7	4.0	1.1
Tyrosine (g)	1.3	3.2	0.7
Valine (g)	2.0	4.8	1.2
Arginine (g)	2.2	5.4	0.9
Histidine (g)	1.1	2.6	0.5
Lysine (g)	3.0	7.2	0.9
**Macrominerals**			
Calcium (g)	1.0	2.5	1.0
Phosphorus (g)	0.9	2.1	0.8
Potassium (g)	1.5	3.6	1.0
Magnesium (g)	0.1	0.3	0.2
Sodium (g)	0.3	0.6	0.2
**Trace elements**			
Zinc (mg)	18	45	15
Iron (mg)	13	31	7.5
Manganese (mg)	1.4	3.4	1.3
Copper (mg)	1.2	2.9	1.5
Selenium (μg)	69	167	88
Iodine^e^ (μg)	0.2	0.5	220
**Essential fatty acids**			
Linoleic acid (g)	1.8	4.4	2.8
Linolenic acid (g)	0.06	0.15	0.11
EPA (g)	0.28	0.68	0.06
DHA (g)	0.22	0.54	0.06
**Vitamins**			
Choline (mg)	530	1300	420
Thiamin (mg)	0.4	1.1	0.6
Riboflavin (mg)	1.2	2.9	1.3
Niacin (mg)	11	26	4.3
Pantothenic acid (mg)	4.1	9.9	3.8
Pyridoxine (mg)	1.0	2.4	0.4
Folate (μg)	67	150	68
Cobalamin (μg)	4.7	11	8.7
Biotin (μg)	20	48	–
Vitamin K (μg)	170	410	410
Vitamin A (RAE^f^, μg)	1.4	3.5	0.7
Vitamin D (μg)	4.0	9.8	3.4
Vitamin E (mg)	27	66	7.5

a *NRC, National Research Council, Recommended Allowance, Nutrient Requirements of Dogs and Cats, National Academy Press, Washington, DC, 2006*.

b *USDA Nutrient Database, United States Department of Agriculture, Agricultural Research Service, Beltsville, MD, https://ndb.nal.usda.gov/ndb/*.

c *Nordic Naturals, Omega-3 PetTM, Watsonville, CA*.

d *Balance-IT® Canine Plus, Davis Medical Consulting, Woodland, CA*.

e *Not listed for all ingredients except for vitamin-mineral supplement*.

f *Retinol activity equivalents expressed in micrograms of all-trans retinol*.

Unexpectedly, the owner reported an absence of SND-associated clinical signs approximately 3 weeks after the diet modification, which also coincided with the last AAI treatment. The AAI was postponed and was next administered 7 weeks later (i.e., 10 weeks from the last treatment). While the dog remained subclinical, an AAI was administered at the owner's request as a preventative measure prior to departing on a vacation with limited access to a veterinary specialist.

The home-prepared diet was well-accepted and the frequency of AAI was maintained (q10–12 weeks) without emergence of SND clinical signs for 520 days (1.4 years). On day 740 the dog presented for evaluation of hyporexia, lethargy, and signs of abdominal pain. Ultrasound imaging and endoscopic biopsy indicated pancreatitis and gastrointestinal inflammation. Appetite improved with medical treatment and feeding of a low fat commercial therapeutic diet (Royal Canin Canine Gastrointestinal Low Fat™ wet). Crusting of the lips developed during the hyporexic period and persisted during feeding of the commercial food. The dog was gradually transitioned back to the homemade diet after 6 weeks and crusting of the lips resolved.

**Figure 3:**
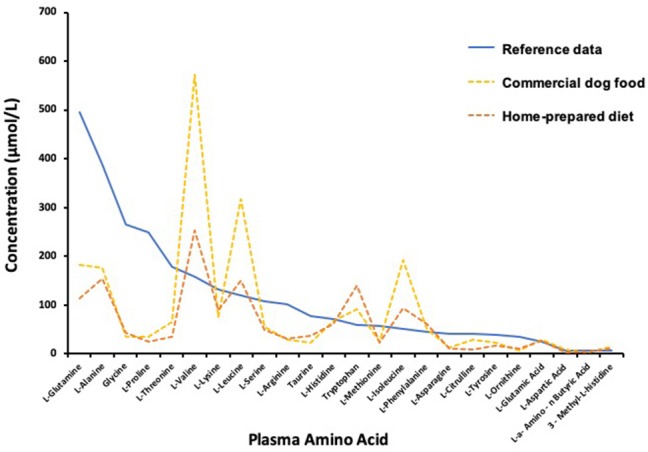
Plasma amino acid concentrations determined 5 h after a morning meal during transition from commercial diet (Day 786) to an owner-prepared, prescribed diet (Day 826) compared to laboratory means reported for normal healthy dogs.

In order to better understand the reason why diet modification alone resulted in substantial clinical improvement and decreased the frequency of AAI, plasma extracted from venous blood collected in a tube containing lithium heparin (56 USP/3 ml, Vacutainer®, Becton, Dickinson and Company, Franklin Lakes, NJ) was obtained before AAI, approximately 5 h after a morning meal, on days 786 and 826. The first sample was collected while eating the commercial low fat food and the second sample collected 4 weeks after transition back to the homemade diet. The samples were stored at −20°C until later shipment on ice packs for analysis in accord with instructions of Amino Acid Laboratory, University of California, Davis, CA (https://www.vetmed.ucdavis.edu/labs/amino-acid-laboratory). Amino acid concentrations in the samples were low relative to the laboratory reference interval with a few notable exceptions (Figure 3). Tryptophan and branched-chain amino acids (BCAA) valine, leucine, and isoleucine were substantially greater than the reference interval concentrations. Concentrations of 25-hydroxyvitamin (OH)- D were 26 and 34 ng/mL in the first and second samples, respectively, where the laboratory mean (± SEM) for apparently healthy dogs (*n* = 57) was 33.9 ± 1.3 ng/mL (RCB, unpublished data).

The dog continued to receive AAI typically once every 10–12 weeks until he was euthanized on day 938 (~2.5 years) for reasons related to severe, refractory *Malassezia* skin infections unrelated to SND.

## Discussion

The etiopathogenesis for the characteristic skin lesions seen in dogs with SND is unclear and likely multifactorial. In people, SND, also known as necrolytic migratory erythema, is most commonly the result of pancreatic glucagonoma; however, there are other pathologies (e.g., inflammatory bowel disease, celiac disease, pancreatic insufficiency, zinc deficiency, and liver disease) associated with the development of the pseudoglucagonoma syndrome (3, 4). In contrast to people, the majority of dogs with SND have nonglucagonoma-related disease associated with an underlying hepatopathy typically of unknown etiology or associated with phenobarbital administration. Diseases that have been reported to occur concurrently with SND in dogs includes diabetes mellitus and hyperadrenocorticism (1, 2, 5–11). The specific pathomechanism of the hepatic form of SND in dogs is unknown but is postulated to occur secondary to metabolic disturbances that result in increased hepatic catabolism of amino acids. Importantly, the aforementioned pathologies reported to occur concurrently in dogs with SND does not suggest causation. At the time of diagnosis, the pancreas appeared normal on contrast-enhanced CT of the abdomen making glucagonoma unlikely.

Hypoaminoacidemia is a hallmark feature of SND in both dogs and people regardless of the underlying cause of disease (i.e., glucagonoma or pseudoglucagonoma syndrome) and as such is suspected to be integral to SND pathogenesis (1, 2, 4, 12). The decreased plasma amino acids found in this dog are consistent with other studies. The amino acid concentrations were low even though samples were collected after a meal during the absorptive phase. Post-prandial compared to over-night, food-deprived amino acid concentrations in plasma better reflect the profile of amino acids derived from food and amino acids that may be limiting in a diet. The low amino acid concentrations were unlikely to be the result of vascular hypoplasia. Tryptophan and BCAA concentrations in the plasma from this dog were greater than the normal reference data mean. The unusual amino acid profile might be a consequence of extraordinary catabolism of amino acids by the liver, as may occur with excessive glucagon stimulation of the liver. Tryptophan and BCAA are mostly catabolized by extrahepatic tissues making their plasma abundance less affected by liver catabolism (2). Muscle is the primary site of BCAA uptake, where leucine serves as a potent initiator of muscle protein synthesis (13). Post-prandial elevation of BCAA may have been especially heightened in this dog because of slow uptake of the amino acids by extrahepatic tissues. Use of BCAA for protein synthesis might have been impaired by insulin resistance and limited availability of circulating amino acids. Diabetes mellitus is associated with insulin resistance and elevation of circulating BCAA (14). This effect appeared blunted on the homemade diet compared to the commercial food (Figure 3), which may indicate better insulin sensitivity and muscle utilization of amino acids with consumption of the prescribed homemade diet.

Sufficient tryptophan concentrations were observed in this dog and other studies that investigated dogs with SND (2). Plasma glucagon was not assessed in this dog. When measured, however, dogs with SND do not commonly have plasma glucagon concentrations greater than the upper limit of the reference interval, which in part may relate to assay limitations (10). Further, approximately 50% of people with pseudoglucagonoma syndrome have plasma glucagon concentrations within the reference interval (15, 16).

Medical management in dogs with SND because of nonglucagonoma-related disease, similar to people with pseudoglucagonoma syndrome, is aimed at treating the underlying condition and repletion of various nutrients including zinc, essential fatty acids, and amino acids (1, 4, 11). Parenteral administration of amino acids is preferred in both people and dogs with SND over the oral route in order to bypass portal circulation and an exaggerated hepatic first pass effect (1, 2, 4). It is clear that parenteral administration of amino acids mitigates the painful clinical signs associated with SND in dogs. However, repeated AAI are costly as they require hospitalization with central-line catheters, or permanent indwelling venous access ports. In this case report, the frequency of AAI was initially based on relapse of clinical signs resulting in an infusion every 2–3 weeks. Importantly, all treatments associated with diabetes mellitus, hyperadrenocorticism, and SND were established and remained unchanged at the time the dog was transitioned to the homemade diet. Diet modification was the only therapeutic variable that changed during the period in which the frequency of AAI decreased from every 2–3 weeks to every 10–12 weeks. It should also be noted that the decision to treat with AAI at 10–12 week intervals was not based on the return of clinical signs; rather, it was centered on owner convenience and a shift in goals to prevent recrudescence of clinical signs. Crusting of the lips did occur after diet change during an episode of pancreatitis. AAI in conjunction with transition back to the homemade diet resolved these signs. Therefore, it is reasonable to suspect diet modification was a large contributing factor for improvement in clinical signs and decreased frequency of AAI in this dog.

The best oral nutritional approach for dogs with SND is unknown, but a high-quality, nutritionally complete, and high-protein diet is recommended except in the presence of hepatic encephalopathy where tolerance to dietary protein is compromised. The diet prior to modifications in this dog was approximately 30% of calories from protein. The initially prescribed homemade diet was approximately 37% of calories from protein. While this is an increase in percent protein, the total caloric intake was intentionally reduced from an estimated 556–411 kcal/day based on the overweight status of the dog and calculated energy needs based on ideal body weight. Consequently, actual dietary protein intake was reduced with the diet change. Therefore, it is unlikely the quantity of protein in the modified diet was important in the observed clinical improvement in this dog.

The diet in this dog prior to the full nutrition assessment varied. It was estimated that only 47% of the caloric intake was composed of a nutritionally complete and balanced commercial dog food. The additional food items, most of which were foods intended for human consumption (raw egg yolk, cheddar cheese, and canned chicken) were high in protein and fat. The formulated homemade diet was simplistic, but some differences in the homemade diet included lack of processing imposed on commercial dog food, balanced to meet all the nutrition requirements of an adult dog, adding egg whites, cooking the eggs, and sweet potatoes. Only 12 amino acids are assessed when using the homemade diet software (Balance IT®, Davis Medical Consulting, Woodland, CA), with lysine being one. The software indicated that the homemade diet abundantly provided lysine, at 791% of the NRC recommended allowance (RA). Recently, extraordinary urinary loss of lysine has been reported for dogs with SND and speculated to be of special importance to SND pathogenesis (12).

The use of eggs in the treatment of dogs with SND is anecdotal. In one study, feeding egg yolks as a supplementation to the diet in 6 dogs had rapid partial to complete reversal of the cutaneous lesions (10). There was no indication if the egg yolk was raw or cooked in that study, nor if the eggs were from chickens or another type of animal. There were only two dogs in the aforementioned study that experienced an increase in plasma amino acids after supplementation and 3 dogs were euthanized at a later point due to complications related to diabetes mellitus. There were no other treatments described for these 6 dogs with SND, nor was the rationale provided as to why egg yolks were supplemented. Egg yolk provides some protein, but more than half of amino acids in egg are in the egg white. Egg yolks have most of the vitamins, such as vitamin A, B_6_, B_12_, D, E, and K. Inclusion of the egg yolks did not change with use of the initially prescribed homemade-diet in the dog reported here, with the exception that they were cooked. Egg whites are substantive sources of niacin, potassium, riboflavin, and magnesium.

Sweet potatoes are high in fiber, vitamin A, and vitamin C, as well as many other vitamins and minerals. The homemade diet formulated for the dog in this report was high in vitamin A retinol activity equivalences (RAE), providing over 1,000% of the NRC RA. Unlike cats, dogs are able to convert carotenoids to vitamin A in the diet, and dietary carotenoids are assumed to have a low toxicity for dogs. The role of vitamin D may also have had an impact on the favorable response to the homemade diet. There was an increase in plasma 25(OH)-D_3_, the major circulating form of vitamin D, after being fed the homemade diet. Most likely, the egg in the diet contributed substantial amounts of dietary Vitamin D_3_. The Balance IT® supplement contains vitamin D_2_. There are increased concentrations of inflammatory mediators (e.g., arachidonic acid, prostaglandins, and leukotrienes) in the epidermis of some people with SND (17). Interestingly, both vitamin A and vitamin D have potent immunomodulatory functions that could have benefited this dog. In people, vitamin A dampens inflammation by shifting CD4+ T-lymphocyte subtypes from T_H_1 and T_H_17 (pro-inflammatory) to T_H_2 and T_Reg_ (anti-inflammatory) (18). Moreover, vitamin D in people and dogs decreases leukocyte production of tumor-necrosis factor (pro-inflammatory) while concomitantly increasing interleukin-10 (anti-inflammatory) cytokines (18–21). Future studies investigating the potential for repletion of vitamin D deficiency in dogs with SND are warranted.

## Conclusion

In conclusion, this report highlights a novel role for nutritionally balanced, prescription, home-made diets with high quality protein to substantially increase the time interval between AAI and improve the outcome for some dogs with SND.

## Data Availability Statement

All datasets generated for this study are included in the article/supplementary material.

## Ethics Statement

All clients (dog owners) at the University of Missouri Veterinary Health Center sign documents at the time of hospital admission that states data from medical records have potential to be used in future publications.

## Author Contributions

JJ, RB, MS, and CR: medical diagnosis, management of case, collection of data, writing and editing manuscript, review of final submission. SF and KS: collection of data, writing and editing manuscript, review of final submission.

### Conflict of Interest

The authors declare that the research was conducted in the absence of any commercial or financial relationships that could be construed as a potential conflict of interest.
